# Ferritin Is a Marker of Inflammation rather than Iron Deficiency in Overweight and Obese People

**DOI:** 10.1155/2016/1937320

**Published:** 2016-12-27

**Authors:** Abidullah Khan, Wazir Muhammad Khan, Maimoona Ayub, Mohammad Humayun, Mohammad Haroon

**Affiliations:** KTH, Peshawar, Pakistan

## Abstract

*Background.* In clinical practice, serum ferritin is used as a screening tool to detect iron deficiency. However, its reliability in obesity has been questioned.* Objectives.* To investigate the role of ferritin in overweight and obese people, either as a marker of inflammation or iron deficiency.* Methods.* On the basis of body mass index (BMI), 150 participants were divided into three equal groups: A: BMI 18.5–25 kg/m^2^, B: BMI 25–30 kg/m^2^, and C: BMI > 30 kg/m^2^. Serum iron, total iron binding capacity (TIBC), transferrin saturation, ferritin, C-reactive protein, and hemoglobin (Hb) were measured for each participant and analyzed through SPSS version 16. One-way ANOVA and Pearson's correlation tests were applied.* Results.* Ferritin was the highest in group C (M = 163.48 ± 2.23, *P* < 0.001) and the lowest in group A, (M = 152.78 ± 1.81, *P* < 0.001). Contrarily to ferritin, transferrin was the lowest in group C, (M = 30.65 ± 1.39, *P* < 0.001) and the highest in group A, (M = 38.66 ± 2.14, *P* < 0.001). Ferritin had a strong positive correlation with both BMI (*r* = 0.86, *P* < 0.001) and CRP (*r* = 0.87, *P* < 0.001) and strong negative correlation with Hb, iron, TIBC, and transferrin saturation (*P* < 0.001).* Conclusion.* Ferritin is a marker of inflammation rather than iron status in overweight and obese people. Complete iron profile including transferrin, rather than serum ferritin alone, can truly predict iron deficiency in such people.

## 1. Introduction

Obesity is a global health problem. In 2014, 600 million adults and 42 million children, less than five years of age, were obese [1]. This is more common in women and is being considered as the most serious threat to human health by different authorities [2]. The normal body mass index (BMI) is 18–25 kg/m^2^. Anyone with a BMI of 25–30 kg/m^2^ is called overweight, while the term “obesity” is used, when BMI exceeds 30 kg/m^2^. This most serious health problem was classified by the American Medical Association (AMA), as a “disease” in 2013 [3].

Overweight and obese people are at risk of developing certain serious complications like obstructive sleep apnea, cor pulmonale, ischemic heart disease, diabetes, and many others [4]. At the same time, it increases the likelihood of iron deficiency (ID) and iron deficiency anemia (IDA), leading to an escalating disease burden [5–9].

Obesity predisposes affected individuals to subclinical inflammation. Many studies have concluded that overweight and obese people are in a state of an ongoing subclinical inflammation that can secondarily lead to more catastrophic events like iron deficiency, malignancy, and so forth [10–13].

Ferritin is used a marker of iron deficiency in various healthcare facilities across the globe [14]. Being an acute phase reactant, serum ferritin level is prone to be higher in overweight and obese people, because of a state of subclinical, but generalized inflammation in them [15–17]. Due to this fact, using serum ferritin as a marker of ID or IDA in overweight or obese people is controversial [16, 17].

Obesity is an emerging health related concern in Pakistan and so are ID and IDA [18–20]. It is also worth mentioning that serum ferritin assay is the most commonly used blood test to diagnose ID or IDA, not only in Pakistan, but in other developing countries of the world also. This is so because there is a relative scarcity of more specific test for the diagnosis of ID or IDA like transferrin saturation or total iron binding capacity (TIBC) and so forth. Therefore, the use and interpretation of serum ferritin assay alone become challenging, because serum ferritin may not be a true measure of an underlying iron deficiency in overweight and obese people. Therefore, this study will focus on correlating serum ferritin with C-reactive protein (CRP) and its association with iron and BMI, and to find out whether, it is safe to keep using serum ferritin as a true measure of iron deficiency in overweight and obese people or not.

## 2. Material and Methods

This cross-sectional, observational study was conducted in the department of medicine of Khyber Teaching Hospital (KTH), Peshawar, Pakistan, between September 2015 and July 2016. This study was approved by the hospital's ethics review committee and included 150 otherwise healthy participants. Informed written consent was obtained from every participant individually.

A structured questionnaire including closed ended questions regarding demographic details, present and past medical/surgical history, any history of recent infections, recent or previous drug history, especially, iron tablets or injections, steroids or oral contraceptive pills, and chemotherapeutic medications like methotrexate, azathioprine, and so forth was used.

**Figure 1:**
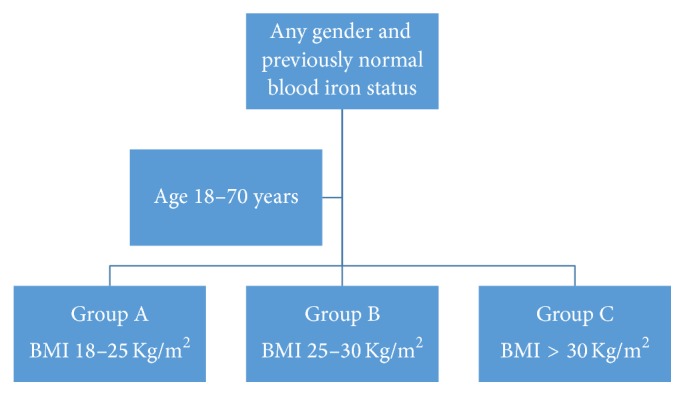
Inclusion criteria for suitability in the study sample.

**Figure 2:**
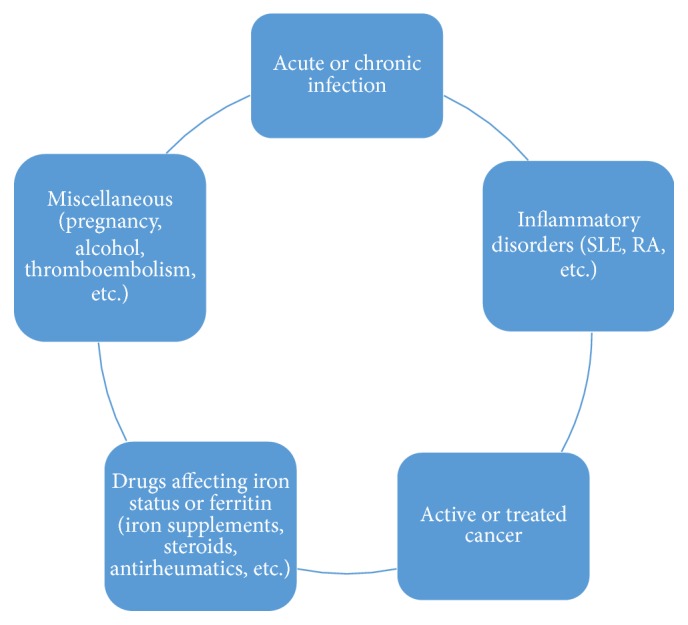
An overview of the chief exclusion criteria.

An overview of the inclusion and exclusion criteria is given below (Figures 1 and 2). The study included patients from both the genders in the age range of 18 to 70 years. Patients already diagnosed with iron deficiency or iron deficiency anemia were excluded from the study. Similarly, those diagnosed with iron overload were also excluded. Other exclusion criteria were set on the basis of medical conditions which could potentially affect the body iron stores or ferritin, such as pregnancy, alcoholism, hemoglobinopathies, diabetes mellitus, bleeding disorders, any acute or chronic inflammatory conditions like rheumatoid arthritis (RA) or systemic lupus erythematosus (SLE), and any acute illnesses during the preceding one month or chronic infections like tuberculosis, brucellosis, and so forth. Patients on drugs which could influence serum iron or ferritin like any use of iron supplements, steroids, oral contraceptive pills (OCPs), nonsteroidal anti-inflammatory drugs (NSAIDS), disease modifying antirheumatic drugs like methotrexate, azathioprine, hydroxychloroquine, and so forth and those with cancer or on active treatment for it were also excluded from the study.

The final 150 individuals included in the study were divided into three groups, each group consisting of 50 participants as follows: group A: BMI 18.5–25 kg/m^2^, group B: BMI 25–30 kg/m^2^, and group C: BMI > 30 kg/m^2^.

The BMI was calculated using the formula weight (kg)/height (meter^2^). A weight scale with a built-in stadiometer was used to measure the weight and height of all the subjects in kilograms and meters, respectively. A morning sample of 5 mL of venous blood was drawn; 3 mL was put in gel tubes for the determination of serum ferritin, iron, total iron binding capacity (TIBC), transferring saturation, and C-reactive protein (CRP), while 2 mL was put in an EDTA tube for hemoglobin (Hb) determination. Serum ferritin and CRP were measured by using enzyme linked immunosorbent assay (ELISA) method, serum iron was measured using enzymatic colorimetric method, and Hb was measured on automated analyzer Sysmex. Transferrin saturation was determined by using the formula; iron/TIBC ×100. All these tests were done by the same technician in the main laboratory of Khyber Teaching Hospital (KTH), Peshawar.

All the data was entered into and analyzed by SPSS version 16. In order to compare the means of different study variables, one-way ANOVA with Turkey-HSD was used. Pearson's correlation test was run to analyze an association of BMI with serum ferritin, CRP, serum iron, TIBC, and transferrin saturation. *P* value of <0.05 was considered as significant.

## 3. Results

Amongst all the participants, 47.3% were males and 52.7% were females. The mean age, body mass index (BMI), and serum ferritin (*μ*g/L) of all the participants were 44.71 ± 6.48, 26.91 ± 3.73, and 158.19 ± 4.82, respectively. Group specific descriptive statistics are given below (Table 1).

The data was tested for the assumptions of homogeneity of variances and normality. One-way ANOVA was used to compare the means of different dependent variables between the three groups, namely, group A, group B, and group C, to find out any impact of obesity on the dependent variables, namely, ferritin, Iron, TIBC, hemoglobin (Hb), and transferrin saturation. Ferritin was the highest in group C (M = 163.46, SD = 2.23, *P* < 0.001), higher in group B (M = 158.32, SD = 1.99, *P* < 0.001), and lowest in group A (M = 152–78, SD = 1.81, *P* < 0.001). However, despite the highest ferritin level in group C (the obesity group), transferrin saturation was the lowest (M = 30.65, SD = 1.39, *P* < 0.001). Similarly, along with transferrin, both Hb and iron were the highest in group A, intermediate in group B, and the lowest in group C (Table 1). The post hoc analysis for these variables was significant at *P* < 0.001 in each group (Table 2). There was a statistically significant difference between the three groups for ferritin (*F* (2,147) = 350.80, *P* < 0.001), hemoglobin (*F* (2,147) = 101.90, *P* < 0.001), and iron (*F* (2,147) = 644.54, *P* < 0.001), respectively. The estimate of effect size (Partial Eta Squared) for the difference of ferritin, iron, and Hb between the three groups was 0.83, 0.86, and 0.58, respectively.

For both CRP and transferrin saturation (TSAT), the assumption of homogeneity of variances was not tenable (*P* < 0.05). However, robust tests for the equality of means were statistically significant (*P* < 0.01 for both Welch and Brown-Forsythe tests). Moreover, one-way ANOVA showed a statistically significant difference between the three groups with regard to CRP and TSAT. The results of one-way ANOVA for CRP and TSAT were *F* (2,147) = 396.87, *P* < 0.001 and *F* (2,147) = 262.87, *P* < 0.001, respectively. CRP was found to be highest in group C (M = 11.46, *SD* = 1.46, *P* < 0.001), higher in group B (M = 7.58, *SD* = 1.01, *P* < 0.001), and the lowest in group A (M = 5.28, SD = 0.73, *P* < 0.001).

Finally a correlation test was run between the BMI and the dependent variables, namely, ferritin, iron, transferrin, TIBC, and CRP (Table 3). It is worth mentioning that both ferritin and CRP correlated positively with BMI (*r* = 0.86, *P* < 0.001 and *r* = 0.89, *P* < 0.001, resp.). However, there was a strong negative correlation between the BMI and the rest of the three variables, namely, iron (*r* = −0.89, *P* < 0.001), TIBC (*r* = −0.87, *P* < 0.001), and TSAT (*R* = −0.84, *P* < 0.001). It is also notable that ferritin had a strong positive correlation with CRP (*r* = 0.87, *P* < 0.01) but a negative one with iron (*r* = −0.89, *P* < 0.001), TIBC (*r* = −0.85, *P* < 0.001), and transferrin saturation (*r* = −0.86, *P* < 0.001). The detailed breakdown is given (Table 4). It must be noted that, for the falling values of iron, TIBC, and TSAT, as one moves upwards from group A to group C, there was a not only a corresponding decline in the level of hemoglobin (Hb), but also a significant increase in the level of ferritin (*P* < 0.001).

## 4. Discussion

Iron deficiency (ID) with or without anemia is common in obese people and has certain mechanisms involved in its pathogenesis mainly a state of subclinical inflammation. The low iron levels in overweight and obese subjects are most probably caused by the inflammatory mechanisms, which arrest iron in the reticuloendothelial system by releasing different inflammatory mediators like cytokines and so forth [21, 22]. Our study showed a positive correlation between ferritin and CRP, signifying an underlying low grade inflammation, leading to subsequent iron deficiency, most probably, because of inflammation mediated iron sequestration in the reticuloendothelial system.

Ferritin is an acute phase reactant and is potentially higher in any infective or inflammatory process, making its careful interpretation necessary in such situations. In a recent study, high levels of ferritin were positively correlated with the risk of metabolic syndrome and obesity [23]. In another study, ferritin was found to be positively associated with CRP [24]. Our findings are consistent with these results. However, in sharp contrast to our statistics, a study by Eftekhari et al. concluded that serum ferritin was lower in obese people [25].

Obesity is one of the biggest health challenges of the 21st century. Association of ID/IDA with obesity doubles the ill health effects of the obesity itself. Though chronic inflammation caused by excess adipose tissue, rather than dietary factors, offers a plausible explanation for ID or IDA in obese people, looking for the existence of both acute and chronic infections, and inflammatory conditions need to be considered as well. Moreover, treating IDA in obese people is another challenge itself, as they tend to respond poorly to iron supplementation [5, 26]. In another recent study, administering both oral iron and ascorbic acid as an enhancer of its absorption from the gut made little difference in correcting ID or IDA in overweight or obese individuals [27].

IDA is most commonly caused by nutritional deficiency. On the other hand, obesity is often caused by overnutrition. Hence, the coexistence of both IDA and obesity in the same person represents the opposite ends of the spectrum of under and overnutrition. However, amongst the different factors responsible for their mutual coexistence like dilutional hypoferremia, poor dietary iron intake, increased iron demands, and/or impaired iron absorption, a chemical called hepcidin was recently found to be the main culprit for ID or IDA in obesity. Hepcidin was found in higher titers in obese and overweight individuals. Hepcidin works by causing subclinical inflammation, decreases iron absorption from the gut, and blunts the effects of iron fortification [28, 29]. Reducing weight may lower hepcidin, which may in return improve the iron stores of the body. Treating obesity through surgical approaches like Roux-en-Y gastric bypass (RYGB) is unfortunately related to iron deficiency also. In a recent Chinese study, iron deficiency and IDA both were found to be extremely frequent after RYGB in obese patients with T2DM at 2-year follow-up [30]. It is therefore recommended to screen overweight and obese people for iron deficiency, both before and after any antiobesity surgical intervention is undertaken.

It is to be stated that the results of our study were according to the expectations of the authors. As is evident from our results, ferritin was not a true indicator of an underlying iron deficiency in our study population of overweight and obese individuals. Rather, ferritin correlated positively with the inflammatory marker, CRP, and BMI, making ferritin a marker of inflammation rather than iron status in overweight and obese people. However, we recommend further studies and would advocate a bigger sample size, in order to arrive at a more logical conclusion.

## 5. Conclusion

Ferritin is a marker of inflammation rather than iron status in overweight and obese individuals. Being an acute phase reactant, a high ferritin level secondary to subclinical inflammation in overweight and obese people may mask an underlying iron deficiency. It is therefore recommended to request a complete iron studies profile including transferrin saturation, ferritin, total iron binding capacity (TIBC), and serum iron, to confidently exclude ID in obese or overweight subjects.

## Figures and Tables

**Table 1:** Descriptive statistics of different study variables in each group.

Variable	Group A (*N* = 50)		Group B (*N* = 50)		Group C (*N* = 50)	
	Mean	SD	Mean	SD	Mean	SD
Age (years)	44.84	6.16	44.84	6.17	44.44	7.17
BMI (kg/m^2^)	22.36	1.08	27.18	0.80	31.18	0.85
Hb (gm/dL)	14.13	0.83	12.68	0.68	11.86	0.88
Iron (*μ*mol/L)	24.86	1.24	21.36	1.04	17.68	1.18
TIBC (*μ*mol/L)	63.56	0.50	59.52	0.86	57.44	1.59
TSAT (%)	38.66	2.14	35.36	1.63	30.65	1.39
Ferritin (*μ*g/L)	152.78	1.81	158.32	1.99	163.48	2.23
CRP (mg/L)	5.28	0.73	7.58	1.01	11.46	1.46

Group A: BMI 18.5–25 kg/m^2^, group B: BMI 25–30 kg/m^2^, and group C: BMI > 30 kg/m^2^, Hb: hemoglobin, TIBC: total iron binding capacity, TSAT: transferrin saturation, CRP: C-reactive protein, and BMI: body mass index.

**Table 2:** Results of post hoc analysis by Turkey HSD.

Dependent variable	(*I*) group	(*J*) group	Mean difference (*I* − *J*)	Std. error	Sig.	95% confidence interval	
						Lower bound	Upper bound
Hb	A	B	1.450^*∗*^	.161	.000	1.07	1.83
		C	2.270^*∗*^	.161	.000	1.89	2.65
	B	A	−1.450^*∗*^	.161	.000	−1.83	−1.07
		C	.820^*∗*^	.161	.000	.44	1.20
	C	A	−2.270^*∗*^	.161	.000	−2.65	−1.89
		B	−.820^*∗*^	.161	.000	−1.20	−.44

Iron	A	B	3.500^*∗*^	.232	.000	2.95	4.05
		C	7.180^*∗*^	.232	.000	6.63	7.73
	B	A	−3.500^*∗*^	.232	.000	−4.05	−2.95
		C	3.680^*∗*^	.232	.000	3.13	4.23
	C	A	−7.180^*∗*^	.232	.000	−7.73	−6.63
		B	−3.680^*∗*^	.232	.000	−4.23	−3.13

Ferritin	A	B	−5.540^*∗*^	.404	.000	−6.50	−4.58
		C	−10.700^*∗*^	.404	.000	−11.66	−9.74
	B	A	5.540^*∗*^	.404	.000	4.58	6.50
		C	−5.160^*∗*^	.404	.000	−6.12	−4.20
	C	A	10.700^*∗*^	.404	.000	9.74	11.66
		B	5.160^*∗*^	.404	.000	4.20	6.12

^*∗*^The mean difference is significant at the 0.05 level.

**Table 3:** Pearson's correlation of BMI with test variables *(note a strong positive correlation with ferritin and CRP)*.

Variable	Pearson's value (*r*)	*P* value
HB	−0.77	<0.001
Iron	−0.89	<0.001
TIBC	−0.87	<0.001
Transferrin	−0.84	<0.001
Ferritin	0.86	<0.001
CRP	0.89	<0.001

**Table 4:** Pearson's correlation of ferritin with the test variables *(note a strong positive correlation with CRP/BMI and an inverse correlation with transferrin)*.

Variable	Pearson's value (*r*)	*P* value
Hb	−0.62	<0.001
Iron	−0.89	<0.001
TIBC	−0.84	<0.001
TSAT	−0.86	<0.001
CRP	0.87	<0.001
BMI	0.86	<0.001

## Abbreviations

ID: Iron deficiency
IDA: Iron deficiency anemia
CRP: C-reactive protein
BMI: Body mass index
TSAT: Transferrin saturation
TIBC: Total iron binding capacity
Hb: Hemoglobin
RYGB: Roux-en-Y gastric bypass.
